# Supplementary material for: Heparin-based hydrogel scaffolding alters the transcriptomic profile and increases the chemoresistance of MDA-MB-231 triple-negative breast cancer cells
Source: Biomater Sci. 2020 Feb 13;8(10):2786–96. doi: 10.1039/c9bm01481k (PMC7497406; doi:10.1039/c9bm01481k)
Supplement: Supplementary file 2 [file BM-008-C9BM01481K-s002.zip › Supplementary File 4/EGFvControl/Pathways/my_analysis.Gsea.1545200981068/HALLMARK_UNFOLDED_PROTEIN_RESPONSE.html]

Details for gene set HALLMARK\_UNFOLDED\_PROTEIN\_RESPONSE[GSEA]

|  || Dataset | expr.class.cls#EGF\_versus\_CONTROL.class.cls#EGF\_versus\_CONTROL\_repos |
| Phenotype | class.cls#EGF\_versus\_CONTROL\_repos |
| Upregulated in class | EGF |
| GeneSet | HALLMARK\_UNFOLDED\_PROTEIN\_RESPONSE |
| Enrichment Score (ES) | 0.38916722 |
| Normalized Enrichment Score (NES) | 1.6509354 |
| Nominal p-value | 0.0032626428 |
| FDR q-value | 0.0057553905 |
| FWER p-Value | 0.068 |
Table: GSEA Results Summary

  

Fig 1: Enrichment plot: HALLMARK\_UNFOLDED\_PROTEIN\_RESPONSE      
 Profile of the Running ES Score & Positions of GeneSet Members on the Rank Ordered List

  

| PROBE | DESCRIPTION (from dataset) | GENE SYMBOL | GENE\_TITLE | RANK IN GENE LIST | RANK METRIC SCORE | RUNNING ES | CORE ENRICHMENT || 1 | PSAT1 | na |  |  | 142 | 2.222 | 0.0189 | Yes |
| 2 | MTHFD2 | na |  |  | 172 | 2.163 | 0.0431 | Yes |
| 3 | SLC7A5 | na |  |  | 213 | 2.074 | 0.0656 | Yes |
| 4 | EXOSC9 | na |  |  | 296 | 1.957 | 0.0845 | Yes |
| 5 | SRPRB | na |  |  | 436 | 1.815 | 0.0988 | Yes |
| 6 | NOLC1 | na |  |  | 443 | 1.809 | 0.1199 | Yes |
| 7 | RRP9 | na |  |  | 522 | 1.757 | 0.1367 | Yes |
| 8 | EXOSC4 | na |  |  | 569 | 1.726 | 0.1547 | Yes |
| 9 | EXOSC2 | na |  |  | 609 | 1.700 | 0.1729 | Yes |
| 10 | EIF4A3 | na |  |  | 702 | 1.642 | 0.1875 | Yes |
| 11 | IARS | na |  |  | 1033 | 1.502 | 0.1881 | Yes |
| 12 | DKC1 | na |  |  | 1144 | 1.464 | 0.1997 | Yes |
| 13 | NOP56 | na |  |  | 1153 | 1.459 | 0.2166 | Yes |
| 14 | HSPA9 | na |  |  | 1253 | 1.427 | 0.2283 | Yes |
| 15 | IFIT1 | na |  |  | 1463 | 1.358 | 0.2335 | Yes |
| 16 | TARS | na |  |  | 1506 | 1.343 | 0.2472 | Yes |
| 17 | GEMIN4 | na |  |  | 1521 | 1.340 | 0.2624 | Yes |
| 18 | TATDN2 | na |  |  | 1526 | 1.339 | 0.2780 | Yes |
| 19 | CEBPG | na |  |  | 1827 | 1.261 | 0.2773 | Yes |
| 20 | SDAD1 | na |  |  | 1945 | 1.236 | 0.2858 | Yes |
| 21 | FKBP14 | na |  |  | 2020 | 1.215 | 0.2964 | Yes |
| 22 | CKS1B | na |  |  | 2048 | 1.207 | 0.3093 | Yes |
| 23 | GOSR2 | na |  |  | 2229 | 1.173 | 0.3138 | Yes |
| 24 | BANF1 | na |  |  | 2269 | 1.162 | 0.3255 | Yes |
| 25 | EXOSC1 | na |  |  | 2478 | 1.117 | 0.3279 | Yes |
| 26 | EXOSC10 | na |  |  | 2591 | 1.095 | 0.3350 | Yes |
| 27 | EIF2S1 | na |  |  | 2819 | 1.058 | 0.3356 | Yes |
| 28 | LSM1 | na |  |  | 2846 | 1.053 | 0.3468 | Yes |
| 29 | FUS | na |  |  | 2941 | 1.036 | 0.3541 | Yes |
| 30 | XPOT | na |  |  | 2983 | 1.029 | 0.3642 | Yes |
| 31 | EIF4E | na |  |  | 3033 | 1.021 | 0.3737 | Yes |
| 32 | KIF5B | na |  |  | 3136 | 1.000 | 0.3803 | Yes |
| 33 | DDX10 | na |  |  | 3349 | 0.962 | 0.3806 | Yes |
| 34 | SKIV2L2 | na |  |  | 3534 | 0.931 | 0.3820 | Yes |
| 35 | NOP14 | na |  |  | 3700 | 0.905 | 0.3841 | Yes |
| 36 | SEC11A | na |  |  | 3889 | 0.870 | 0.3845 | Yes |
| 37 | SPCS3 | na |  |  | 4135 | 0.831 | 0.3816 | Yes |
| 38 | EIF4A2 | na |  |  | 4370 | 0.798 | 0.3788 | Yes |
| 39 | KHSRP | na |  |  | 4535 | 0.773 | 0.3794 | Yes |
| 40 | SERP1 | na |  |  | 4992 | 0.709 | 0.3639 | Yes |
| 41 | PDIA6 | na |  |  | 5001 | 0.708 | 0.3719 | Yes |
| 42 | EIF4EBP1 | na |  |  | 5073 | 0.697 | 0.3764 | Yes |
| 43 | IMP3 | na |  |  | 5216 | 0.676 | 0.3770 | Yes |
| 44 | EXOSC5 | na |  |  | 5294 | 0.666 | 0.3809 | Yes |
| 45 | STC2 | na |  |  | 5348 | 0.658 | 0.3859 | Yes |
| 46 | ALDH18A1 | na |  |  | 5433 | 0.646 | 0.3892 | Yes |
| 47 | POP4 | na |  |  | 5661 | 0.615 | 0.3846 | No |
| 48 | DCP2 | na |  |  | 5954 | 0.576 | 0.3761 | No |
| 49 | NFYB | na |  |  | 5979 | 0.572 | 0.3817 | No |
| 50 | EIF4A1 | na |  |  | 6111 | 0.553 | 0.3814 | No |
| 51 | H2AFX | na |  |  | 6160 | 0.547 | 0.3853 | No |
| 52 | PREB | na |  |  | 6354 | 0.520 | 0.3814 | No |
| 53 | LSM4 | na |  |  | 6619 | 0.490 | 0.3734 | No |
| 54 | SHC1 | na |  |  | 6751 | 0.474 | 0.3721 | No |
| 55 | SLC30A5 | na |  |  | 6811 | 0.466 | 0.3746 | No |
| 56 | CNOT4 | na |  |  | 7273 | 0.411 | 0.3553 | No |
| 57 | SPCS1 | na |  |  | 7349 | 0.402 | 0.3562 | No |
| 58 | ATF3 | na |  |  | 7410 | 0.395 | 0.3577 | No |
| 59 | EXOC2 | na |  |  | 7491 | 0.386 | 0.3581 | No |
| 60 | RPS14 | na |  |  | 7647 | 0.369 | 0.3543 | No |
| 61 | PARN | na |  |  | 7748 | 0.357 | 0.3533 | No |
| 62 | ATP6V0D1 | na |  |  | 7887 | 0.338 | 0.3501 | No |
| 63 | CXXC1 | na |  |  | 8151 | 0.310 | 0.3400 | No |
| 64 | SLC1A4 | na |  |  | 8321 | 0.291 | 0.3346 | No |
| 65 | DNAJA4 | na |  |  | 8389 | 0.285 | 0.3345 | No |
| 66 | ARFGAP1 | na |  |  | 8494 | 0.272 | 0.3323 | No |
| 67 | TUBB2A | na |  |  | 8831 | 0.234 | 0.3175 | No |
| 68 | NFYA | na |  |  | 8841 | 0.232 | 0.3198 | No |
| 69 | ATF6 | na |  |  | 9376 | 0.176 | 0.2939 | No |
| 70 | CNOT6 | na |  |  | 9422 | 0.167 | 0.2935 | No |
| 71 | TTC37 | na |  |  | 9767 | 0.129 | 0.2770 | No |
| 72 | PDIA5 | na |  |  | 9856 | 0.121 | 0.2738 | No |
| 73 | SSR1 | na |  |  | 10012 | 0.105 | 0.2670 | No |
| 74 | YIF1A | na |  |  | 10156 | 0.087 | 0.2605 | No |
| 75 | EDC4 | na |  |  | 11642 | -0.076 | 0.1836 | No |
| 76 | PAIP1 | na |  |  | 11673 | -0.081 | 0.1830 | No |
| 77 | WFS1 | na |  |  | 11974 | -0.122 | 0.1688 | No |
| 78 | ATF4 | na |  |  | 12198 | -0.144 | 0.1588 | No |
| 79 | DCP1A | na |  |  | 12216 | -0.145 | 0.1596 | No |
| 80 | SEC31A | na |  |  | 12524 | -0.187 | 0.1458 | No |
| 81 | ASNS | na |  |  | 12529 | -0.188 | 0.1478 | No |
| 82 | CNOT2 | na |  |  | 12914 | -0.234 | 0.1305 | No |
| 83 | DCTN1 | na |  |  | 13434 | -0.306 | 0.1069 | No |
| 84 | ZBTB17 | na |  |  | 13611 | -0.332 | 0.1016 | No |
| 85 | HYOU1 | na |  |  | 13838 | -0.358 | 0.0940 | No |
| 86 | DNAJC3 | na |  |  | 14042 | -0.384 | 0.0879 | No |
| 87 | BAG3 | na |  |  | 14949 | -0.507 | 0.0465 | No |
| 88 | CALR | na |  |  | 15148 | -0.538 | 0.0425 | No |
| 89 | CCL2 | na |  |  | 15276 | -0.560 | 0.0425 | No |
| 90 | EIF2AK3 | na |  |  | 15524 | -0.598 | 0.0367 | No |
| 91 | NHP2 | na |  |  | 15612 | -0.608 | 0.0393 | No |
| 92 | ERN1 | na |  |  | 15686 | -0.624 | 0.0429 | No |
| 93 | CEBPB | na |  |  | 15897 | -0.665 | 0.0398 | No |
| 94 | XBP1 | na |  |  | 16003 | -0.687 | 0.0424 | No |
| 95 | HSP90B1 | na |  |  | 16095 | -0.702 | 0.0460 | No |
| 96 | HSPA5 | na |  |  | 16140 | -0.715 | 0.0522 | No |
| 97 | WIPI1 | na |  |  | 16225 | -0.738 | 0.0565 | No |
| 98 | EDEM1 | na |  |  | 16338 | -0.765 | 0.0598 | No |
| 99 | CHAC1 | na |  |  | 16392 | -0.779 | 0.0662 | No |
| 100 | IGFBP1 | na |  |  | 16692 | -0.855 | 0.0607 | No |
| 101 | KDELR3 | na |  |  | 17046 | -0.961 | 0.0536 | No |
| 102 | HERPUD1 | na |  |  | 17679 | -1.177 | 0.0345 | No |
| 103 | DDIT4 | na |  |  | 17752 | -1.202 | 0.0450 | No |
| 104 | TSPYL2 | na |  |  | 17932 | -1.291 | 0.0509 | No |
| 105 | DNAJB9 | na |  |  | 17933 | -1.292 | 0.0663 | No |
Table: GSEA details [plain text format]

  

Fig 2: HALLMARK\_UNFOLDED\_PROTEIN\_RESPONSE      
 Blue-Pink O' Gram in the Space of the Analyzed GeneSet

  

Fig 3: HALLMARK\_UNFOLDED\_PROTEIN\_RESPONSE: Random ES distribution      
 Gene set null distribution of ES for **HALLMARK\_UNFOLDED\_PROTEIN\_RESPONSE**
